# Effectiveness and safety of ultra-slow full power shockwave lithotripsy compared to mini–percutaneous nephrolithotomy and retrograde intrarenal surgery for treatment of lower calyceal stone between 1 and 2 cm with high attenuation value

**DOI:** 10.1007/s00345-025-06004-6

**Published:** 2025-11-11

**Authors:** Mahmoud Abdallah, Mohammad Talaat Mohammad, Ahmed M. Ragheb, Akrm A. Elmarakbi, Ossama Mahmoud

**Affiliations:** 1 Urology department, Faculty of medicine, Beni-Suef University, Beni-Suef, Egypt; 2 Urology department, Sidnawy Insurance hospital, Cairo, Egypt

**Keywords:** Lower calyceal stone, Ultraslow shockwave lithotripsy (ultraslow SWL), Mini-percutaneous nephrolithotomy (Mini-PNL), Retrograde intrarenal surgery (RIRS), Stone-free rate (SFR)

## Abstract

**Purpose:**

This prospective randomized study compared the effectiveness and safety of ultra-slow full power shockwave lithotripsy (ultraslow SWL), mini-percutaneous nephrolithotomy (mini-PNL), and retrograde intrarenal surgery (RIRS) for 1–2 cm lower calyceal stone with HU above 1000.

**Methods:**

360 patients were randomized with stratification according to stone size using a block randomization to: (1) Ultraslow SWL; (2) Mini-PNL; or (3) RIRS. Primary outcomes were stone-free rates (SFR) and complications with blinding of assessors. Our follow-up protocol featured a non-contrast CTUT after 3 months for Ultraslow SWL while 1 month for both mini-PNL and RIRS groups, confirming stone-free status. The SFR was stratified as follow: true stone-free (0 mm), residual fragments ≤ 2 mm, residual fragments 3–4 mm, and failure (more than 4 mm). Secondary outcomes included operative parameters and hospitalization. Multinominal logistic regression was used for significant findings to identify predictors of success or failure.

**Results:**

Mini-PNL showed superior SFR (95%) versus RIRS (85.8%) and SWL (76.7%) (*p* = 0.007). Mini-PNL showed the lowest rates of residual stones (0.8% for fragments ≤ 2 mm, 0.8% for fragments 3–4 mm, and 3.3% for failure), while SWL had the highest residual stone (5% for fragments ≤ 2 mm, 3.3% for fragments 3–4 mm, and 15% for failure), with RIRS demonstrating intermediate outcomes (3.3% for fragments ≤ 2 mm, 3.3% for fragments 3–4 mm, and 7.5% for failure). All techniques had comparable (*p* = 0.110) Clavien Dindo I-II complications (pain, vomiting, colic, hematuria, skin ecchymosis, and fever/UTI) and managed conservatively with analgesics, hydration, or antibiotics, with no major complications (Grade III–V). However, interpretation of comparative outcomes must be cautious due to methodological limitations such as differing follow-up timing.

**Conclusion:**

Mini-PNL achieved higher SFR, its invasiveness and radiation exposure warrant consideration. However, the comparison between RIRS and ultraslow SWL success suggests ultraslow SWL may benefit patients with moderately sized lower pole stones with high attenuation values, especially who wish to avoid invasive surgery or are considered unfit for anesthesia. The results are encouraging but preliminary and require confirmation. However, some limitations were heterogeneous follow-up imaging intervals, absence of anatomical data, lack of stone composition analysis, and baseline stone volume imbalance. Therefore, future studies should evaluate long-term outcomes and cost-effectiveness, and consider our limitations.

## Introduction

Renal stones affect 10–15% of the global population, with higher prevalence in the Middle East and North America due to dietary and climatic factors [1, 2]. Calcium oxalate stones constitute 70–80% of cases, while uric acid and struvite stones are associated with metabolic disorders and UTIs, respectively [3, 4]. The condition causes significant morbidity, including colic, obstruction, and recurrent infections, necessitating effective management strategies [5, 6].

For 1–2 cm lower calyceal stones, three minimally invasive approaches are employed. SWL fragments stones non-invasively but has variable effectiveness and often requires multiple sessions [7, 8]. Based on the available literature data, the SFR for SWL is around 46–64% [9]. PNL achieves higher single-session success (70–95%) but carries risks of bleeding and organ injury [10, 11]. RIRS balances effectiveness (70–90%) and safety, particularly for complex anatomy [12, 13].

Treatment selection hinges on stone characteristics (size, density), renal anatomy, and patient comorbidities [14, 15]. SWL is affected by lower pole anatomy [16], while PNL’s invasiveness may deter use in high-risk patients [17]. RIRS offers versatility but demands technical expertise [18]. Recent meta-analyses highlight ongoing debates about optimal modality selection for 1–2 cm stones [19, 20]. In case of SWL Multiple studies have shown that the success of stone fragmentation declines as the Hounsfield units (HU) rises (>970 HU) [21–24]. Compared to harder stones, stones with a density of less than 970 HU had a much higher chance of successfully undergoing SWL treatment (98% vs. 38%) [21, 25, 26].

According to previous study, ultraslow full-power SWL for high attenuation value stones is associated with an improved stone-free rate without affecting safety [27]. The aim of the study was to compare the effectiveness and safety between ultraslow SWL, mini-PNL and RIRS in managing 1–2 cm lower calyceal stone with HU above 1000.

## Patients and methods

### Study design and setting

This prospective randomized clinical study enrolled 360 patients with 1–2 cm radio-opaque lower calyceal stone (density ≥ 1000 Hounsfield units) from Beni-Suef University Hospital urology department over 36 months. The study protocol was submitted to the ethical committee and ethical approval was obtained from the Faculty of Medicine, Beni-Suef University Research Ethical Committee before the study began to ensure patient confidentiality with approval number (FMBSUREC/06062021/Ali).

### Participants

Participants were consecutively allocated into three groups: ultraslow SWL (Group 1, *n* = 120), mini-PNL (Group 2, *n* = 120), and RIRS (Group 3, *n* = 120). Inclusion criteria included adults aged 18–70 years and < 30 BMI with solitary radio-opaque lower calyceal stone ranging from 1 to 2 cm with stone density ≥ 1000 HU as measured by Non contrast CTUT; exclusion criteria encompassed renal anomalies, prior renal surgery, coagulopathy, active UTI, transplanted kidney, renal insufficiency, or pregnant women.

### Sample size calculations

Sample size was calculated using OpenEpi (v3), with 95% confidence intervals and 90% power. Based on preliminary data and existing literature, the effect size is 0.15. The calculation yielded a required sample size of 120 participants per group (360 participants in total).

### Randomization

All participants were thoroughly informed about the three treatment options during the consent process, including their respective benefits, risks, and expected outcomes. Patients were clearly told they would be randomly allocated to one of the three treatment arms as part of the study protocol. The consent form explicitly stated that treatment assignment would be determined by randomization rather than patient or physician preference. Additionally, the study protocol allowed for crossover to alternative treatments if the initial approach failed, which was explained to all participants during consent.

The randomization was done using computer generated with 1:1:1 ratio using a block randomization design (block size of 6). The randomization was done by an independent biostatistician who was not involved in patient recruitment, treatment, or follow-up. Given the important prognostic influence of stone burden, the randomization was stratified by stone size (10–15 mm vs. 15–20 mm). This ensured a balanced distribution of these critical covariates across the three treatment groups. Treatment assignments were concealed using sequentially numbered, opaque, sealed envelopes, which were opened only after written consent and baseline assessments were completed. Each envelope contained the assigned group on a card inside. Upon obtaining written informed consent and confirming eligibility, the attending urologist responsible for enrollment, would open the next sequentially numbered envelope in the presence of the patient to reveal the group assignment.

**Fig. 1:**
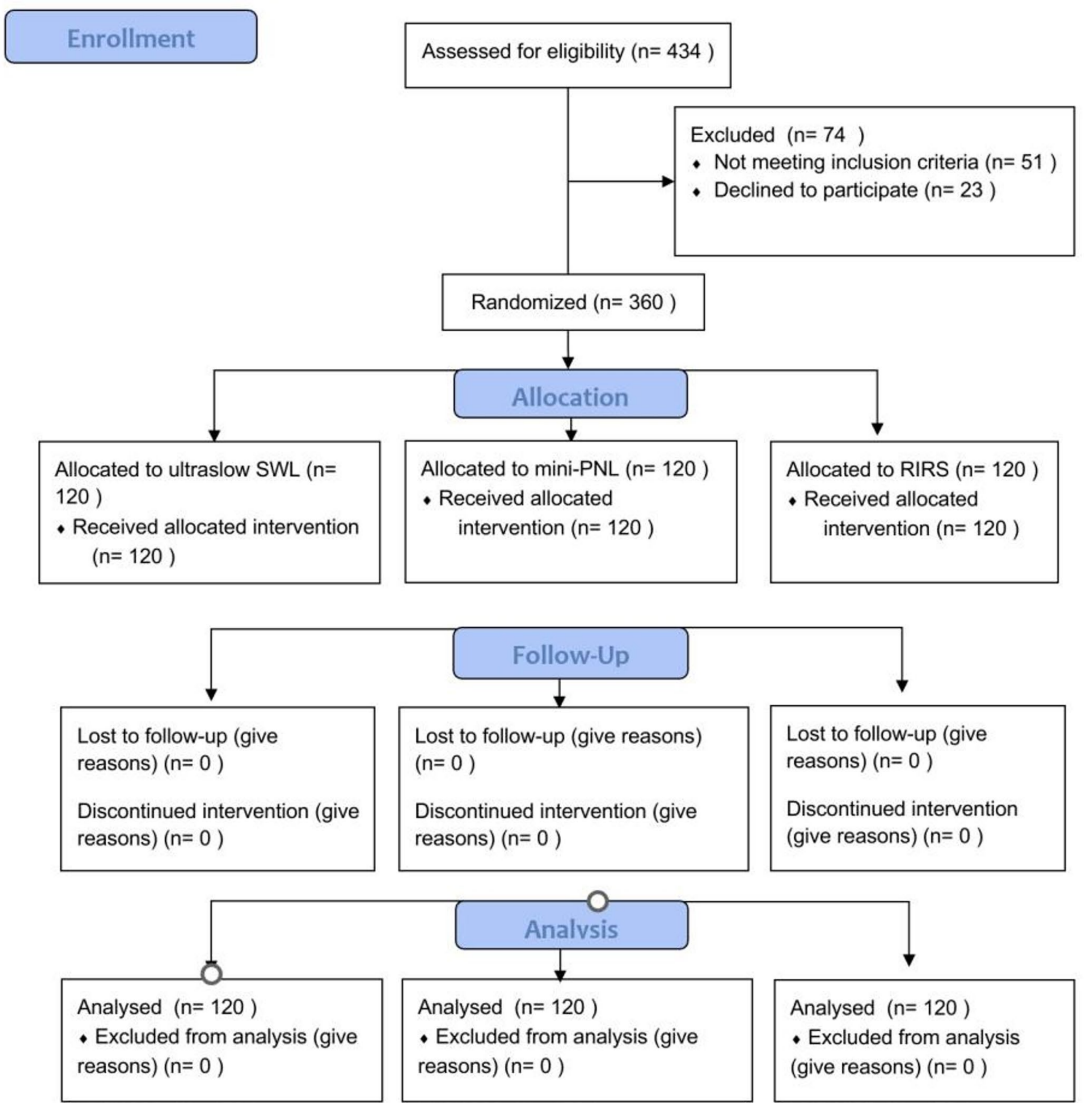
CONSORT flow diagram

A CONSORT flow diagram are shown in Fig. 1 to clearly depict the number of participants in each stage of the trial (Fig. 1).

### Interventions

All procedures were performed by experienced consultants in our university with more than 10 years of experience. The three groups’ procedures were as follow:

#### Group 1 (Ultra-slow full power SWL)

Patients received IV meperidine hydrochloride (1 mg/kg) analgesia before treatment with an EMD E-1000 electrohydraulic lithotripter (focal zone 2.4 × 0.6 cm, depth 13.5 cm). Using real-time fluoroscopic guidance, we implemented a novel dual-phase ramping protocol: initial escalation from 6 to 18 kV over 100 shocks (2 min pause), followed by 18–22 kV over 100 shocks (2 min pause), then maintained at the therapeutic level of 22 kV, the device’s maximum output [27]. The ultraslow rate of 30 shocks/minute (total 2500–3000/session) combined with strategic pauses significantly reduced renal injury risk while maintaining efficacy. Post-procedure monitoring included 2-hour observation for hematuria or colic, with discharge criteria requiring pain-free voiding. Our follow-up protocol featured serial ultrasound (48 h, 2 weeks) to detect complications and KUB X-ray at 2 weeks assessing fragmentation, and non-contrast CTUT at 3 months confirming stone-free status. Patients received tamsulosin 0.4 mg daily and analgesics as needed, with repeat sessions (max 3) for persistent fragments ≥ 4 mm.

#### Group 2 (mini-PNL)

After antibiotic prophylaxis and general anesthesia, patients underwent initial lithotomy position, ureteral catheter placement (6Fr) for retrograde pyelography. In prone position with careful padding, we achieved precise calyceal access using an 18G needle under fluoroscopic guidance. The tract was dilated to 18Fr using sequential dilators, maintaining a safety wire throughout. Through a 15Fr nephroscope (Richard Wolf), we performed complete stone clearance using a Swiss LithoClast Master at 12 Hz under direct vision. Our tubeless protocol included antegrade 6Fr Double-J stent and urethral catheter. Postoperative care emphasized early ambulation and renal ultrasound at 24 h to exclude silent hemorrhage and catheter removed after 2 days. Our follow-up protocol for this group featured serial ultrasound (48 h, 2 weeks) to detect complications or residual fragments and non-contrast CTUT at 1 month confirming stone-free status and to do a Double-J stent removal.

#### Group 3 (RIRS)

Following IV antibiotic prophylaxis and general anesthesia, procedures were performed in lithotomy position. After cystoscopic guide wire placement, we performed gradual ureteral dilation to 14 Fr using Teflon dilators under fluoroscopy before deploying a 11/13 Fr access sheath. Using a digital flexible ureteroscope (Boston Scientific LithoVue 9.5 Fr), we employed a standardized dusting technique with 100 W Holmium laser (200 μm fiber) at low energy/high frequency (0.5 J, 20 Hz) until complete stone pulverization. The primary lithotripsy mode was dusting, defined as a technique with the clinical goal of ablating the stone into fine, passable particles (< 1 mm) to minimize the need for basket extraction and facilitate spontaneous passage. To achieve this, we employed a low-energy/high-frequency setting of 0.5 J and 20 Hz (10 W). This combination provides a high rate of thermal ablation while minimizing retropulsion, thereby allowing for efficient and rapid surface vaporization of the stone to create a cloud of fine debris. A double-J stent wasn’t placed preoperative for safety and efficacy [28]; however, a 6Fr Double-J stent was routinely placed for 4 weeks, with Foley catheter removal postoperatively after one day after confirming absence of hematuria. Our technique emphasized minimizing intrarenal pressures through intermittent irrigation and sheath use. Our follow-up protocol for this group featured serial ultrasound (48 h, 2 weeks) to detect complications or residual fragments and non-contrast CTUT at 1 month confirming stone-free status and to do a Double-J stent removal.

All patients with unsuccessful initial treatment (failed stone fragmentation or inability to access the stone) underwent a secondary procedure to ensure complete stone resolution. For example, in RIRS cases where the lower pole stone could not be reached, patients were offered either mini-PNL or ultraslow SWL, depending on stone characteristics and patient preference. These subsequent interventions ensured that all patients ultimately achieved stone-free status, and no untreated cases remained.

### Data collection

Preoperative evaluation included history, stone size in mm, stone density in HU, and preoperative hemoglobin. To avoid the inter-observer variability, HU was measured by doing non-contrast CT and a blinded urologist with a blinded radiologist measures the HU as a double reading using a standardized protocol: the region of interest was placed centrally within the stone on non-contrast CT, avoiding adjacent tissues or artifacts, and the mean of three consecutive measurements was recorded [29]. Since blinding of the surgeons performing the procedures was not feasible due to the nature of the interventions. Both the urologist and the radiologist were blinded to the patient’s treatment allocation for the study groups to minimize detection bias. Intraoperative data included procedure duration (minutes), and radiation exposure time (seconds). Postoperative assessment focused on postoperative hemoglobin, hospital stay in days, catheterization time in days, and complications of each procedure classified by the Clavien-Dindo system. Hematuria was recorded based on visible macroscopic hematuria observed post-procedure and confirmed by urinalysis when persistent beyond 24 h. Pain was evaluated qualitatively based on patients’ self-reported experience during and immediately after the procedure, and was categorized as present or absent. SFR is defined as complete clearance on imaging at follow up. We stratified the SFR as follow: true stone-free (0 mm), residual fragments ≤ 2 mm, residual fragments 3–4 mm, and failure (more than 4 mm).

### Statistical analyses of data

Data were analyzed using SPSS v26 (SPSS Inc., Chicago, IL, USA). Continuous non-normally distributed data (confirmed by Kolmogorov-Smirnov test) were reported as median (IQR) and analyzed using Kruskal-Wallis test with post hoc analysis for significant variables. Categorical data were presented as frequencies (%) and assessed with Chi-square tests, followed by multinominal logistic regression for significant findings. A p-value ≤ 0.05 was considered statistically significant.

## Results

### Baseline characteristics

The study included a total of 360 patients, evenly distributed into three groups of 120 patients each, undergoing ultraslow SWL, mini-PNL, and RIRS. Table 1 shows that the median ages of the patients were 43.50 years in the ultraslow SWL group, 37 years in the mini-PNL group, and 36.50 years in the RIRS group, with no statistically significant difference in age (*p* = 0.172). The BMI showed no statistically significant difference across the groups (*p* = 0.145). The majority of patients in all three groups were male, with 75% in ultraslow SWL, 69.2% in mini-PNL, and 61.7% in RIRS, though the difference in sex was not statistically significant (*p* = 0.083). Stone size, stone volume, stone density, and preoperative hemoglobin levels were comparable across the groups, with no significant differences (*p* = 0.815, *p* = 0.419, *p* = 0.223, and *p* = 0.969, respectively).

### Outcome measures

#### Post operative hemoglobin levels

In terms of outcome measures, postoperative hemoglobin levels were significantly lower in the mini-PNL group (median 11.9) compared to the ultraslow SWL (median 12.75) and RIRS (median 12.7) groups (*p* < 0.001). Post hoc analysis was done to the statistically significant variables. The pairwise comparison between the mini-PNL and RIRS groups yielded an adjusted significance value of 0.003. Similarly, the mini-PNL and ultraslow SWL groups showed a significant difference, with an adjusted p-value of 0.001. However, the comparison between RIRS and ultraslow SWL resulted in a non-significant difference (adjusted p-value = 1.000).

### Operative time

Operative time was longest in the ultraslow SWL group (median 105 min), followed by mini-PNL (median 80.50 min) and RIRS (median 76 min), with a statistically significant difference (*p* < 0.001). The mean sessions of ultraslow SWL were 1.608 sessions. The pairwise comparison in the post hoc analysis between the mini-PNL and RIRS groups yielded an adjusted significance value of 0.02. Similarly, both the RIRS & ultraslow SWL groups and mini-PNL & ultraslow SWL showed a significant difference, with an adjusted p-value lower than 0.001.

### Radiation exposure time

Radiation exposure time was highest in the mini-PNL group (median 128 s) and lowest in the RIRS group (median 33 s), with ultraslow SWL falling in between (median 68.50 s), and this difference was also statistically significant (*p* < 0.001). The pairwise comparison in the post hoc analysis between the RIRS & ultraslow SWL, mini-PNL & RIRS, and ultraslow SWL & mini-PNL yielded an adjusted significance value lower than 0.001.

### Hospital stay in days

Hospital stay was shortest in the ultraslow SWL group (median 0.21 days), while both mini-PNL and RIRS had a median hospital stay of 1 day (*p* < 0.001). The pairwise comparison in the post hoc analysis between both the mini-PNL& ultraslow SWL and RIRS & ultraslow SWL groups yielded an adjusted significance value lower than 0.001. However, the comparison between RIRS and mini-PNL resulted in a non-significant difference (adjusted p-value = 1.000).

### Catheter time in days

No Catheter was inserted in the ultraslow SWL group (median 0 days), longer in the RIRS group (median 1 day), and longest in the mini-PNL group (median 2 days), with a statistically significant difference (*p* < 0.001). The pairwise comparison in the post hoc analysis between the RIRS & ultraslow SWL, mini-PNL & RIRS, and ultraslow SWL & mini-PNL yielded an adjusted significance value lower than 0.001.

### Success rate

Complication rates were similar across the groups, with no significant difference in the distribution of Grade 1 and Grade 2 complications (*p* = 0.110). Overall, Grade I complications (pain, vomiting, hematuria, colic, skin ecchymosis) varied according to the procedure. Hematuria and pain were the most frequent events across all groups, particularly common after mini-PNL and RIRS, while colic and vomiting were less frequent and mostly associated with ultraslow SWL and RIRS. Skin ecchymosis was observed almost exclusively after ultraslow SWL, reflecting its characteristic extracorporeal nature. All Grade 1 complications were managed conservatively with analgesics, antiemetic, electrolytes, hydration, and observation, without the need for further intervention. On the other hand, grade 2 complications (fever/UTI) were managed by antipyretics and antibiotics according to culture and sensitivity. No Grade III–V complications occurred, supporting the safety of all three modalities.

However, the success rate, defined as being stone-free, was highest in the mini-PNL group (95%), followed by RIRS (85.8%) and ultraslow SWL (76.7%), with a statistically significant difference (*p* = 0.007). Residual fragments ≤ 2 mm were observed in 6 patients (5%) after SWL, 1 patient (0.8%) after mini-PNL, and 4 patients (3.3%) after RIRS. Residual stones measuring 3–4 mm occurred in 4 patients (3.3%) each in the SWL and RIRS groups, while only 1 patient (0.8%) was reported in the mini-PNL group. Failure of fragmentation was most frequent with SWL, occurring in 18 patients (15%), compared to 9 patients (7.5%) with RIRS and only 4 patients (3.3%) with mini-PNL. The post hoc analysis shows that the comparison between both the mini-PNL & RIRS and mini-PNL & ultraslow SWL groups yielded an adjusted significance value lower than 0.001. However, the comparison between RIRS & ultraslow SWL resulted in a non-significant difference (adjusted p-value = 0.085).

Auxiliary procedures were presented to failed patients within 4 to 6 weeks. As for the 18 patients who failed in ultraslow SWL, 10 preferred RIRS while 8 preferred mini-PNL. Regarding the 4 patients who failed in mini-PNL, 3 patients preferred to ultraslow SWL while only one patient preferred RIRS. Additionally, from the 9 patients who failed in RIRS, 5 preferred ultraslow SWL and 4 preferred mini-PNL.

**Table 1:** Comparisons between the groups (*n* = 360)

Variable	Ultraslow-SWL	Mini-PNL	RIRS	*p* value
	(*n* = 120)	(*n* = 120)	(*n* = 120)	
*Baseline characteristics*				
Age, years (median (IQR))	43.50 (24)	37 (22)	36.50 (22)	0.172
*Sex*				
Male (n (%))	90 (75%)	83 (69.2%)	74 (61.7%)	0.083
Female (n (%))	30 (25%)	37 (30.8%)	46 (38.3%)	
BMI, kg/m^2^ (median (IQR))	27 (3)	26 (4)	27 (5)	0.145
Stone size, mm (median (IQR))	13 (5)	14 (4)	14 (5)	0.815
Stone volume, mm^3^(median (IQR))	732 (553)	906 (492)	906 (614)	0.419
Stone density, HU (median (IQR))	1259.50 (219)	1248 (238)	1275 (232)	0.223
Preoperative Hg (median (IQR))	12.8 (2)	12.6 (2.1)	12.8 (2.2)	0.969
*Outcome measures*				
Postoperative Hg (median (IQR))	12.75 (1.94)	11.9 (2)	12.7 (2.1)	**< 0.001**
Operative time, minutes (median (IQR))	105 (106)	80.50 (14)	76 (13)	**< 0.001**
Radiation exposure time, sec (median (IQR))	68.50 (65)	128 (46)	33 (5)	**< 0.001**
Hospital stay, days (median (IQR))	0.21 (0.20)	1 (0)	1 (0)	**< 0.001**
Catheter time, days (median (IQR))	0 (0)	2 (0)	1 (0)	**< 0.001**
*Complications*				
Grade 1 (n (%))	101 (84.2%)	93 (77.5%)	105 (87.5%)	0.11
Pain	30 (29.7)	25 (26.9%)	35 (33.3%)	
Vomiting	5 (5%)	8 (8.6%)	10 (9.5%)	
Colic	20 (19.8%)	3 (3.2%)	15 (14.3%)	
Hematuria	35 (34.7%)	40 (43.0%)	35 (33.3%)	
Skin ecchymosis	11 (10.8%)	2 (2.2%)	10 (9.5%)	
Grade 2 (n (%))				
Fever and UTI	19 (15.8%)	27 (22.5%)	15 (12.5%)	
*Success rate*				
Stone free (n (%))	92 (76.7%)	114 (95%)	103 (85.8%)	**0.007**
Residual ≤ 2 mm	6 (5%)	1 (0.8%)	4 (3.3%)	
Residual 3–4 mm	4 (3.3%)	1 (0.8%)	4 (3.3%)	
Failed not fragmented (n (%))	18 (15%)	4 (3.3%)	9 (7.5%)	

*IQR* Interquartile range; *n* number; % percentage

Bolded p value denotes significance

Table 2 shows that multinomial logistic regression analysis, with “Stone free” as the reference category, revealed several significant predictors for the different success rate outcomes. The model’s pseudo-R-squared value was 0.107. Treatment modality was the only independent predictor of treatment failure. Other covariates (age, sex, stone size, stone volume, and stone density) were not significant predictors.

For the outcome of Failed, compared to being Stone free, the type of lithotripsy treatment was a significant predictor. Specifically, patients who underwent ultraslow SWL had significantly higher odds of failure (Odds ratio (OR) = 5.131, *p* = 0.004) compared to those who were Stone free. Similarly, RIRS was also significantly associated with increased odds of failure (OR = 3.299, *p* = 0.045) when compared to the Stone free outcome. No other variables showed a statistically significant association with the outcomes of “Residuals ≤ 2 mm” or “Residuals 3–4 mm” when compared to “Stone free”.

**Table 2:** Multinominal logistic regression of the succession rate predictors

Parameter Estimates					
Success rate^a^	B	Sig.	Exp(B)	95% confidence interval for Exp(B)	
				Lower bound	Upper bound
*Residuals ≤ 2 mm*					
Intercept	0.289	0.959			
Age	−0.031	0.162	0.970	0.929	1.012
Stone size (mm)	0.306	0.552	1.358	0.495	3.723
Stone density (HU)	−0.005	0.535	0.895	0.891	0.991
Stone volume (mm^3^)	−0.001	0.616	0.999	0.997	1.002
Ultraslow SWL	0.641	0.345	1.898	0.502	7.176
RIRS	0.356	0.630	1.428	0.335	6.083
Mini-PNL	0^b^				
Female	−0.510	0.452	0.601	0.159	2.270
Male	0^b^				
Grade 1 complications	0.236	0.768	1.266	0.265	6.056
Grade 2 complications	0^b^				
*Residuals 3–4 mm*					
Intercept	−12.233	0.132			
Age	0.011	0.623	1.012	0.966	1.059
Stone size (mm)	0.860	0.315	2.364	0.442	12.658
Stone density (HU)	0.000	0.982	1.000	0.996	1.004
Stone volume (mm^3^)	−0.003	0.274	0.997	0.992	1.002
Ultraslow SWL	0.751	0.398	2.118	0.371	12.094
RIRS	0.728	0.414	2.071	0.361	11.894
Mini-PNL	0^b^				
Female	−0.038	0.957	0.962	0.234	3.950
Male	0^b^				
Grade 1 complications	0.693	0.519	1.999	0.243	16.438
Grade 2 complications	0^b^				
*Failed*					
Intercept	−7.996	0.034			
Age	0.005	0.730	1.005	0.979	1.031
Stone size (mm)	0.372	0.301	1.451	0.717	2.934
Stone density (HU)	0.000	0.774	1.000	0.998	1.003
Stone volume (mm^3^)	−0.001	0.270	0.999	0.997	1.001
Ultraslow SWL	1.635	**0.004**	5.131	1.663	15.828
RIRS	1.194	**0.045**	3.299	1.027	10.601
Mini-PNL	0^b^				
Female	0.172	0.659	1.188	0.552	2.557
Male	0^b^				
Grade 1 complications	0.770	0.225	2.160	0.622	7.503
Grade 2 complications	0^b^				

*Exp(B)* Odds ratio (OR)

Bolded p value denotes significance

^a^The reference category is: Stone free

^b^This parameter is set to zero because it is redundant

## Discussion

This study compared the outcomes of ultraslow SWL, mini-PNL, and RIRS for treating 1–2 cm lower calyceal stone in 360 patients. Our findings align with the broader literature while providing nuanced insights into the trade-offs between effectiveness, invasiveness, and recovery. The SFR was highest for mini-PNL (95%), followed by RIRS (85.8%) and ultraslow SWL (76.7%), with statistically significant differences. Although our post hoc analysis revealed no statistically significant difference between RIRS and ultraslow SWL, suggesting encouraging effectiveness in achieving stone-free status.

These results corroborate meta-analyses by Donaldson et al. [19] and Bozzini et al. [30], which consistently ranked PNL and RIRS above SWL for lower pole stones. However, our ultraslow SWL success rate was notably higher than the 50–70% range reported in older studies [31], possibly are due to using ultraslow SWL in the management of stones with a high attenuation value which result in very small fragments facilitating their expulsion.

Stratified SFR analysis reveals critical prognostic differences. Mini-PNL’s superior true stone-free (0 mm) rate offers the lowest long-term risk. While RIRS and ultraslow SWL achieved good overall SFR, a larger proportion of their successes involved residual fragments. Fragments ≤ 2 mm can be managed conservatively but retain a risk of future events. Crucially, fragments 3–4 mm are associated with a significantly higher risk of symptoms, complications, and secondary procedures [13, 32]. Accordingly, we emphasize that stratifying SFR into true stone-free, ≤ 2 mm, and 3–4 mm residuals is clinically meaningful and should guide patient counseling and follow-up. Thus, ultraslow SWL’s tendency to leave these larger fragments may deter its use for patients seeking a definitive, single-session outcome.

The ultraslow full-power was at rate of 30 shock waves/min in which a previous study by Al-Dessoukey et al. [27] showed that ultraslow full-power SWL for high attenuation value stones is associated with an improved stone-free rate without affecting safety. This may explain the encouraging outcomes of the ultraslow SWL group with the higher success rate. The shockwave delivery rate significantly impacts SWL success, with slower rates demonstrating superior stone fragmentation [33]. Another previous porcine study by Paterson et al. [34] showed 30 SWs/min reduced large fragments (>2 mm) by 45% versus 120 SWs/min (from 81% to 45%). Additionally, Kang et al.‘s meta-analysis (13 studies) confirmed clinically, with low-frequency SWL (60–70/min) yielding higher SFRs (OR 2.2, 1.5–2.6) than high-frequency (100–120/min) [35].

This can be explained that slower shock wave delivery rates allow both the stone and surrounding tissues to recover slightly between pulses, which contributes to more effective lithotripsy. This brief recovery period can reduce stone movement, enabling the energy to remain more accurately focused on the target. It also minimizes interference from cavitation bubbles, which can otherwise dissipate the shock wave energy, and helps limit tissue damage, thereby improving the overall safety and efficiency of the treatment [36].

The effectiveness of ultraslow SWL for dense stones arises from three synergistic mechanisms. First, the reduced shockwave rate allows complete cavitation bubble collapse between pulses, generating more potent secondary shockwaves that target the stone’s structural integrity. Second, sustained full-power energy deposition creates thermal stress through differential expansion of stone components, progressively weakening the crystalline lattice. Third, the slower rate inherently reduces tissue injury by minimizing cumulative trauma, enabling safer delivery of higher total energy to resistant stones. Together, these effects overcome the inherent challenge of fragmenting high-density calculi while preserving renal function [36, 37].

The combination of a low shockwave rate and the use of a wide focal zone potentially allows for more efficient energy dissipation and reduced shear stress on renal parenchyma, thereby minimizing the risk of vascular injury and hematoma formation compared to conventional higher-rate protocols [36, 37]. However, the primary clinical relevance of our findings is that this ultraslow SWL protocol presents itself as a viable, non-invasive treatment option for select 1–2 cm lower pole stones, offering a safety profile comparable to endoscopic techniques while preserving its non-invasive advantage.

Operative times in our cohort were longest for ultraslow SWL (median 105 min) compared to RIRS (76 min) and mini-PNL (80.5 min). This contrasts with some studies [27] where SWL was quicker (26–49 min), likely due to differences in fragmentation efficiency as ultraslow-SWL with slower rates needs longer time and demonstrate superior stone fragmentation as explained. However, operative time of both RIRS and mini-PNL seems higher compared to other studies. A previous study [38] showed that mean operative time was comparable between RIRS and mini-PNL with a slightly non-significant advantage for RIRS (45.8 vs. 52.8 mm). This inconsistency came from the fact that our strict adherence to detailed intraoperative safety steps, including meticulous irrigation control and stepwise tract dilation for mini-PNL, may have added to the operative time, and the larger stone burden in our cohort compared to the cited study may also partly explain the longer duration. Additionally, our study were performed in an academic teaching hospital setting, where consultants have to guide and explain steps for the residents, which tends to prolong operative duration.

Radiation exposure time was significantly higher for mini-PNL (128 s) than RIRS (33 s), a predictable finding given PNL’s reliance on fluoroscopy, as noted by Schoenthaler et al. [39]. RIRS’s minimal radiation aligns with its endoscopic approach. Hospital stay and catheterization times further differentiated the modalities: ultraslow SWL patients were discharged within 0.21 days with no catheter, whereas RIRS and mini-PNL required median stays of 1 day, with catheter durations of 1 and 2 days, respectively. These findings are consistent with the study by Soliman et al. [40], who emphasized SWL’s outpatient advantage but cautioned about its higher retreatment rates.

Our study utilized a dusting technique (0.5 J/20 Hz) for RIRS, aiming to produce fine fragments for spontaneous clearance. However, it is important to note that, as highlighted in a recent systematic review by Moretto et al. [41], there is a notable lack of consensus within endourology regarding the precise definition, laser parameters, and even the clinical endpoints of the ‘dusting’ technique [41]. Parameters described in the literature vary widely in energy and frequency to achieve different goals, from true dusting to fragmenting or a ‘pop-dusting’ hybrid approach. Our choice of 0.5 J/20 Hz represents a commonly reported and practical dusting setting that prioritizes efficiency and safety for stones in the 1–2 cm range. The findings of Moretto et al. [41] underscore the need for future standardized protocols to better compare outcomes across studies. In our study, this technique proved effective, yielding a high SFR with a low complication rate, supporting its utility as a valid approach for lower pole stones.

Complications were statistically comparable across groups (*p* = 0.110), though mini-PNL had a more pronounced hemoglobin drop, consistent with study by Kumar et al. [31] which reported 4% transfusion rates for PNL. SWL’s safety profile in our study contrasts with data on steinstrasse (4–7%) and sepsis risks [42], underscoring the importance of patient selection. Our results showed that hematuria and pain rates appeared higher compared than previous study [43]. This may be due to the fact that our protocol involved delivering full-power shockwaves at ultraslow frequency, which—while effective—may transiently increase renal parenchymal stress compared to conventional protocols, and our systematic follow-up actively captured even mild, self-limiting hematuria and pain episodes, which may have led to higher reporting. Additionally, our study assessed pain qualitatively, relying on patient self-report rather than a standardized numerical scale. This approach may have led to higher recorded rates of pain compared to studies using quantitative measures, where only moderate to severe pain above a certain threshold is usually reported. The qualitative assessment is inherently more sensitive, as any level of discomfort is captured, which may explain the apparent discrepancy with the previous study [43]. Importantly, all events in our study were mild (Clavien-Dindo grade I–II) and resolved conservatively.

Notably, logistic regression revealed no significant predictors of SFR except for modality of treatment. This diverges from a previous study [44] findings, who identified infundibular anatomy as critical for SWL success, suggesting that our cohort’s unselected anatomy may have blunted SWL outcomes. RIRS’s consistent performance—even without anatomical exclusions—supports its role in complex anatomy or high-risk patients, as highlighted in a previous study by Sabnis et al. [18].

This study’s conclusions are specific to 1–2 cm lower calyceal stones. However, for complex cases not studied here (e.g., staghorn calculi, significant anatomical anomalies, or very large stone burdens), alternative treatments are vital. Standard percutaneous nephrolithotomy (PCNL) is the gold standard for large (>2 cm) and complex stones due to high single-session stone-free rates [45]. Furthermore, emerging techniques like robotic-assisted pyelolithotomy may offer a minimally invasive alternative for select complex cases, particularly those with concomitant UPJ obstruction, providing anatomical reconstruction alongside stone clearance [46]. Treatment choice must be guided by stone characteristics, renal anatomy, available expertise, and patient preference.

The study’s limitations include potential selection bias, as we did not exclude unfavorable anatomy, unlike the study by Bozzini et al. [30]. A key limitation is the absence of formal anatomical assessment of the lower pole (e.g., infundibulopelvic angle, infundibular width), which are established predictors of SWL success and fragment clearance. This omission means our outcomes, particularly for ultraslow SWL and RIRS, may be influenced by an uneven distribution of favorable and unfavorable anatomies. Second, although not statistically significant, a numerical imbalance in median stone volume was observed at baseline, with the ultraslow SWL group having a lower stone volume than the mini-PNL and RIRS groups. Given the well-established inverse relationship between stone volume/burden and treatment success, particularly for SWL, this imbalance may have introduced a bias favoring the outcomes in the SWL group. This is an important limitation when interpreting the comparative efficacy results.

Third, as pre-operative stone composition was not available for the majority of patients, we were unable to analyze its impact on treatment outcomes. This is a significant limitation, particularly for interpreting the results of the ultraslow SWL group, as the efficacy of shockwave lithotripsy is profoundly influenced by stone composition. Although we used high attenuation value (> 1000 HU) as a surrogate marker for likely composition, it remains an imperfect proxy. The absence of this data prevents a more nuanced analysis of the factors contributing to treatment success and failure across all modalities.

Fourth, our study protocol featured an inconsistency in the timing of follow-up imaging between the groups. Based on standard clinical practice at our institution for post-procedural monitoring, patients undergoing mini-PCNL and RIRS typically receive their first follow-up NCCT at 1 month to assess for residual fragments or early complications, and to do a Double-J stent removal. In contrast, for ultraslow SWL, a 3-month follow-up is standard to allow adequate time for fragment passage, allowing for additional sessions if needed. This discrepancy introduces a potential systematic bias, as the SWL group had a longer period for spontaneous clearance of small residual fragments before assessment. Therefore, the reported SFR for the SWL group may be favorably influenced by this extended time window compared to the endoscopic groups. This limits the direct comparability of the SFR outcomes and is a significant weakness of the present study.

However, our data robustly affirm that ultraslow SWL has encouraging results compared to RIRS for 1–2 cm lower pole stone, with mini-PNL offering the highest SFR. For SWL, patient counseling about retreatment remains essential. Future studies should standardize anatomical assessments to refine treatment algorithms further to better predict outcomes and tailor therapy. Future studies comparing lithotripsy techniques should prioritize the routine collection of stone composition data, either through pre-operative dual-energy CT or, more reliably, post-operative infrared spectroscopy analysis of retrieved fragments. This would allow for a more detailed and accurate subgroup analysis and provide stronger, composition-specific clinical recommendations. Future prospective, comparative studies must ensure a standardized imaging protocol with identical follow-up time points for all treatment arms to enable unbiased comparison.

## Conclusion

Our study demonstrates that mini-PNL achieves the highest SFR (95%) for 1–2 cm lower calyceal stone, followed by RIRS (85.8%) and SWL (76.7%), with each modality presenting distinct advantages. However, ultraslow SWL and RIRS showed no statistically significant difference in SFR. Our results showed that ultraslow SWL may be an encourging alternative for patients with moderately sized lower pole stones with high attenuation values, especially who wish to avoid invasive surgery or are considered unfit for anesthesia. The novel SWL protocol (ultra-slow full power) appears particularly suitable for dense stones, warranting further comparative studies. The results are encouraging but preliminary and require confirmation. However, some limitations were heterogeneous follow-up imaging intervals, absence of anatomical data, lack of stone composition analysis, and baseline stone volume imbalance. Therefore, future studies should evaluate long-term outcomes and cost-effectiveness, and consider our limitations to further refine clinical decision-making.

## Data Availability

The datasets used and/or analyzed during the current study are available from the corresponding author on reasonable request.
